# The Danish Model for Improvement of Diabetes Care in General Practice: Impact of Automated Collection and Feedback of Patient Data

**DOI:** 10.1155/2012/208123

**Published:** 2012-07-24

**Authors:** Henrik Schroll, René dePont Christensen, Janus Laust Thomsen, Morten Andersen, Søren Friborg, Jens Søndergaard

**Affiliations:** ^1^The Danish Quality Unit of General Practice, Institute of Public Health, University of Southern Denmark, 5000 Odense, Denmark; ^2^The Research Unit of General Practice, Institute of Public Health, University of Southern Denmark, 5000 Odense, Denmark; ^3^Centre for Pharmacoepidemiology, Department of Medicine, Solna, Karolinska Institute, Clinical Epidemiology Unit T2, 17177 Stockholm, Sweden

## Abstract

*Background*. Sentinel Data Capture is an IT program designed to collect data automatically from GPs' electronic health record system. Data include ICPC diagnoses, National Health Service disbursement codes, laboratory analysis, and prescribed drugs. Quality feedback reports are generated individually for each practice on the basis of the accumulated data and are available online only for the specific practice. *Objective*. To describe the development of the quality of care concerning drug prescriptions for diabetes patients listed with GPs using the Data Capture module. *Methods*. In a cohort study, among 8320 registered patients with diabetes, we analyzed the change in the proportion of medication for uncontrolled cases of diabetes. *Results*. From 2009 to 2010, there was an absolute risk reduction of 1.35% (0.89–1.81: *P* < 0.001) in proportion of persons not in antidiabetic medication despite an HbA1c above 7.0. Similarly, there was a 4.51% (3.42–5.61: *P* < 0.001) absolute risk reduction in patients not in antihypertensive treatment despite systolic blood pressure above 130 mm Hg and 4.73% (3.56–5.90: *P* < 0.001) absolute risk reduction in patients with total cholesterol level above 4.5 mmol/L and not receiving lipid-lowering treatment. *Conclusions*. Structured collection of electronic data from general practice and feedback with reports on quality of care for diabetes patient seems to give a significant reduction in proportion of patients with no medical treatment over one year for participating GPs. Due to lack of a control group, we are, however, not able to say if the drop in the proportion of uncontrolled cases is a result of participation in collection of electronic data and feedback alone.

## 1. Background

Several interventions have been investigated in order to improve quality of diabetes care. Two recent systematic reviews evaluated interventions to improve diabetes management including provider education, point-of-care reminders, audit and feedback, and registries. Both reviews showed improved processes for physician-directed interventions but no improvements in patient outcomes for diabetes [1, 2]. Only few studies have, however, dealt with automatically collected and electronic feedback with data to doctors on diabetes care [3].

Danish general practice is considered leading in integrating IT technology in everyday patient care and in the electronic communication with the rest of health care system [4]. In 2006, Danish general practitioners (GPs) were invited to implement a Data Capture module. Data from the participating practices were automatically sent to the National Danish General Practice Database (DAMD). In return, the DAMD provided feedback to the GPs with information on each patient's treatment and quality of care.

The aim of this is to describe the development of the quality of care concerning drug prescriptions for diabetes patients listed with GPs by use of the Data Capture module.

## 2. Material and Methods

### 2.1. Primary Health Care in Denmark

In Denmark health care, contacts are free of charge for the patients and primary health care is organized with self-employed general practitioners (GPs) on contract with 70% pay per fee and 30% pay per capitation. GPs work in singlehanded practices or in cooperation in clinics with 2 or more GPs. Patients must register with a GP or partnership practice of their choice practicing within 15 km of their home and each GP has about 1,600 patients. GPs are gatekeepers of the Danish National Health Service and almost all referrals to specialists and hospitals are made through them.

### 2.2. The Data Capture Module and the DAMD Database

**Figure 1:**
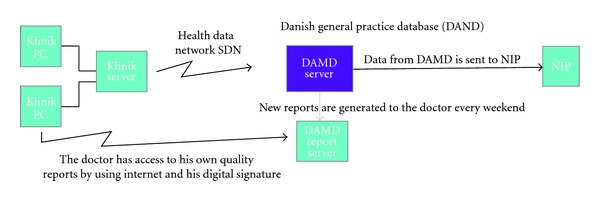
The Sentinel Data Capture program collects key data as they enter into the GP's EMR. The collected data are *prescribed drugs*, National Health Service *disbursement codes*, *laboratory analysis results* and *ICPC diagnoses*. In addition it is possible via pop-up screens to collect data for specific “projects” including chronic diseases and special designed research projects. Every night data are sent to DAMD where updated quality reports are generated every weekend.

**Figure 2:**
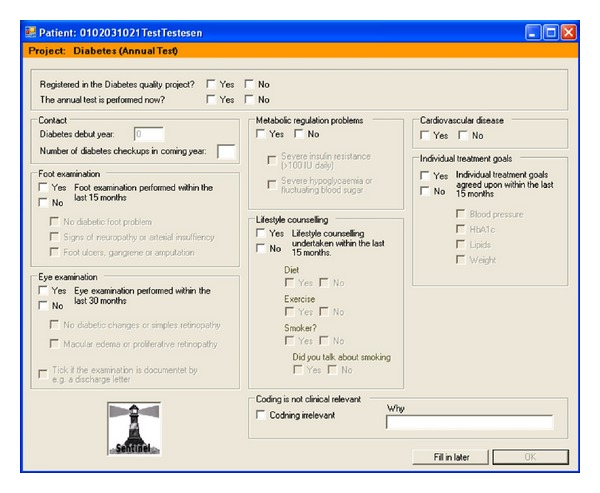
The diabetes popup is displayed once a year on the screen, when the GP enters an ICPC diagnosis for diabetes in his electronic patients files system.

Figure 1 shows the data structure in a clinic where the Data Capture module is installed. Data collected automatically comprise all drug prescriptions, all diagnoses of patient contacts, all disbursement codes, and all laboratory data recorded in the patient file. Once a year a popup will appear on the GPs screen (Figure 2). The popup records data not otherwise available in the electronic journal system, that is, the GP can fill in further nonstructured information regarding diabetes care, for example, information on diet, exercise and smoking habits, and whether or not the patient had been to the ophthalmologist and chiropodist.

**Figure 3:**
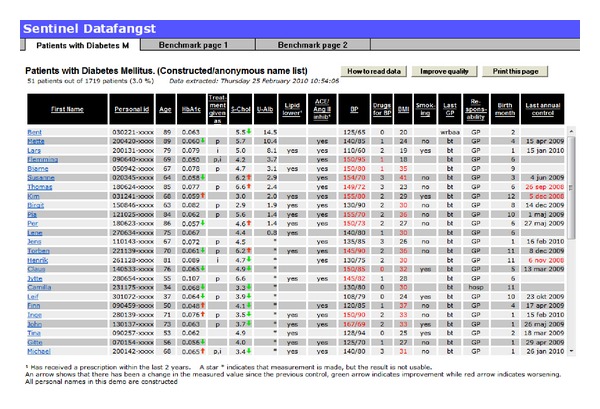
The diabetes feedback report shown when the GP logs into the report server. The GP can see that he has 51 patients with diabetes in his clinic. By clicking in the black area he can sort and rank his patients. For example, by clicking on HBA1c the population will be sorted so the patients with the highest value will come in first line. By clicking on “Benchmark page 1” the GP can compare his performance in Diabetes care with that of his colleagues in the municipality and at national level.

**Figure 4:**
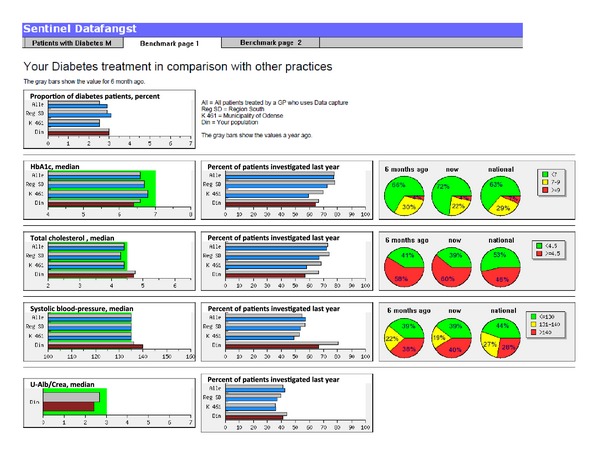
The GPs have access to online data on the report server concerning quality of care indicators from their own practice and they can benchmark the practice against their colleagues at the municipal, regional, and national levels. Figure 4 shows an example with some quality indicators for diabetes care.

Updated quality reports are generated several times a week and made available for the GPs on the report server, where GPs can access their own data from the clinic online by using the professional digital signature. The GPs have access to updated data on the quality of care from their own practice, and they can identify individual patients that are suboptimal treated (Figure 3) and benchmark the practice against their colleagues at the municipal, regional, and national levels (Figure 4). In 2009, 196 clinics used the Data Capture module and with the national contract from April 2011 every Danish GP is obliged within two years to participate in the Data Capture module program and has feedback reports available.

### 2.3. Outcomes and Statistics

We classified patients diagnosed with type 2 diabetes into two groups: controlled and uncontrolled diabetes. The criteria for diagnosis and controlling type 2 diabetes are according to the guidelines by the Danish College of General Practitioners [5]. Patients with type 2 diabetes and a HbA1c above 7.0 mmol/L should be treated with at least one antidiabetic medication, patients with a total cholesterol above 4.5 mmol/L should be treated with lipid-lowering drugs, and all patients with a blood pressure of more than 130 systolic should be treated with antihypertensive. We only included patients with at least two diabetes recordings with at least one year's interval, indicating the patients had been attending at least to yearly diabetes controls. We analyzed change in the proportion of uncontrolled cases concerning Hba1c, cholesterol, and blood pressure. Additionally, we calculated transition of patients from controlled to uncontrolled and vice versa. Data were analyzed using Stata. Differences were assessed using chi-square tests with a two-sided significance level of 0.05. To account for the repeated measurements and clustering at the GP level, we performed a mixed effects, multilevel logistic regression assuming the patients to be nested in relation to the GPs.

## 3. Results

In all, there were 500.606 patients in DAMD in October 2009 and 14.173 patients were diagnosed with type 2 diabetes before October 2009 and alive during the observation period. The number of included patients for each of the three parameters was: 7988 with two HbA1c measurements, 5805 patients with two blood pressure measurements, and 7123 patients with two cholesterol measurements.

 In October 2009, 235 persons registered as diabetics with two HbA1c measurements were not in antidiabetic medication despite an HbA1c above 7.0. After one year, 185 of these were now treated according to guidelines, and only 77 of the patients with treatment or HbA1c below 7.0 in 2009 were in 2010 without treatment despite an HbA1c level above 7.0, resulting in a total of 127 persons not receiving antidiabetic medication despite an HbA1c level above 7.0 in 2010. In total, there was a statistically significant reduction in the proportion of persons not on antidiabetic medication despite an HbA1c above 7.0 from 2.94% in 2009 to 1.59% in 2010 (*P* < 0.001) (Table 1). Concerning lipid-lowering medication, 1226 patients were in 2009 not in lipid-lowering treatment despite total cholesterol above 4.5 mmol/L. In 2010, 532 of these were treated according to guidelines and only 195 went from treatment or cholesterol below 4.5 mmol/L in 2009 to no lipid-lowering treatment despite a value of total cholesterol above 4.5 mmol/L in 2010, giving a statistically significant reduction of cases from 17.21% in 2009 to 12.48% in 2010 (*P* < 0.001). For blood pressure regulation, 722 patients were not on medical antihypertensive therapy despite a systolic blood pressure above 130 mm Hg in 2009. After one year, 385 of these were now treated according to guidelines and only 123 patients went from treatment or systolic blood pressure below 130 mm Hg to no medical antihypertensive therapy despite a systolic blood pressure above 130 mm Hg, resulting in a total of 460 patients with high blood pressure not in medical treatment in 2010 and giving a total reduction of uncontrolled cases from 12.44% in 2009 to 7.92% (*P* < 0.001) in 2010 (Table 1). Multilevel analyses confirmed the significant reduction in the number of uncontrolled cases for both lipid-lowering treatment and antihypertensive treatment (*P* < 0.001 in all three cases).

## 4. Discussion

The main finding is that implementation of the Data Capture module and electronic feedback of qualitative indicators seems to be associated with improvement of the quality of care for patients with type 2 diabetes when studying quality of drug prescriptions. However, these results should be interpreted with caution due to the lack of a control group, as the observed changes could also be attributed to, for example, time trends. The treatment of diabetes patients in general in Denmark is monitored by the Danish National Indicator Project [6], and from the national reports on diabetes from 2011 there was no indication of a general improvement in diabetes care from 2009 to 2011.

Another Danish study [7] on electronic feedback for GPs with regard to treatment status of patients with type 2 diabetes found significantly better medical treatment according to guidelines in the randomized intervention group compared to the control group. That study used an earlier version of the report system than the one in the present study. The results from the present study are in agreement with the results from the randomized study from Guldberg and colleagues, supporting the fact that there may be a promising role for data capture and data feedback as a tool for quality development in general practice. Furthermore, similar results have been found in studies from the USA [8, 9]. Financial incentives increased clinical quality scores for diabetes in the UK [10], but a recent Cochrane review found insufficient evidence to support or not support the use of financial incentives to improve the quality of primary health care [11].

Difficulties with proper coding and registration of diabetic patients may be a weakness in this study, where a possible obstacle to the proper coding could be the fear that persons other than the doctor himself might gain insight into his quality of treatment. In the service agreement for the Data Capture module, only the physician and the individual patient had access to view their own data. When more than 4 clinics are pooled, activities can be published but not in a way that it is possible to identify either the patient or the individual doctor. Data from DAMD are in general anonymous for research purposes. However, to specific quality and research projects it may be possible to obtain access to DAMD data. The researcher or quality development person has to seek permission to use data from DAMD from a quality and research committee comprising medical quality representatives from the 5 regions and representatives from the 3 university research units in Denmark.

The use of diagnosis coding is voluntary in Denmark. Throughout the country, numerous courses in diagnostic coding have been carried out. A national ICPC background group is in charge of developing courses in diagnostic coding and setting up rules for the way diagnosis coding should be perceived and used as clinically relevantly as possible. In a Danish and an Australian study of vignettes, it appeared that in the coding of chronic disease the interobserver agreement was higher (over 70%), while for single contacts and symptom diagnoses the coding was lower (below 70%) [12, 13]. Since then, lectures have been held across the country on ICPC coding. In the ICPC-2 version of the coding system, inclusion and exclusion terms are in use [14, 15], but the ICPC-2 is not implemented in all systems. Once this has happened, coding quality should be followed up with new research. 

## 5. Conclusion

In this uncontrolled study, structured collection of electronic data from general practice and feedback with quality reports seem to reduce the proportion of type 2 diabetes patients with no medication, despite values for HbA1c, blood pressure, and cholesterol levels above target levels. Data capture and electronic feedback of quality indicators might be a promising tool for quality improvement in general practice.

## Figures and Tables

**Table 1:** Number and proportion of patients with no medication despite clinical condition indicating need for treatment now and one year ago among the included type 2 diabetes patients (7988 with recorded HbA1c, 7123 with recorded cholesterol levels, and 5805 with recorded blood pressure).

	Oct. 2009, *N* (%)	Oct. 2010, *N* (%)	Absolute risk reduction (95% CI)
Diabetes control (HbA1c > 7% and no medical treatment)	235 (2.94)	127 (1.59)	1.35% (0.89, 1.81)
Cholesterol (>4.5 mmol/L and no medical treatment)	1226 (17.21)	889 (12.48)	4.73% (3.56, 5.90)
Blood pressure (systolic > 130 and no medical treatment)	722 (12.44)	460 (7.92)	4.51% (3.42, 5.61)
